# Regulating Thermogalvanic Effect and Mechanical Robustness via Redox Ions for Flexible Quasi-Solid-State Thermocells

**DOI:** 10.1007/s40820-022-00824-6

**Published:** 2022-03-25

**Authors:** Peng Peng, Jiaqian Zhou, Lirong Liang, Xuan Huang, Haicai Lv, Zhuoxin Liu, Guangming Chen

**Affiliations:** 1 College of Materials Science and Engineering, Shenzhen University, Shenzhen, 518055 People’s Republic of China; 2 College of Physics and Optoelectronic Engineering, Shenzhen University, Shenzhen, 518060 People’s Republic of China

**Keywords:** Thermocells, Thermoelectric, Flexible energy devices, Wearable applications, Hydrogel electrolytes

## Abstract

The design of power supply systems for wearable applications requires both flexibility and durability. Thermoelectrochemical cells (TECs) with large Seebeck coefficient can efficiently convert low-grade heat into electricity, thus having attracted considerable attention in recent years. Utilizing hydrogel electrolyte essentially addresses the electrolyte leakage and complicated packaging issues existing in conventional liquid-based TECs, which well satisfies the need for flexibility. Whereas, the concern of mechanical robustness to ensure stable energy output remains yet to be addressed. Herein, a flexible quasi-solid-state TEC is proposed based on the rational design of a hydrogel electrolyte, of which the thermogalvanic effect and mechanical robustness are simultaneously regulated via the multivalent ions of a redox couple. The introduced redox ions not only endow the hydrogel with excellent heat-to-electricity conversion capability, but also act as ionic crosslinks to afford a dual-crosslinked structure, resulting in reversible bonds for effective energy dissipation. The optimized TEC exhibits a high Seebeck coefficient of 1.43 mV K^−1^ and a significantly improved fracture toughness of 3555 J m^−2^, thereby can maintain a stable thermoelectrochemical performance against various harsh mechanical stimuli. This study reveals the high potential of the quasi-solid-state TEC as a flexible and durable energy supply system for wearable applications.

**Supplementary Information:**

The online version contains supplementary material available at 10.1007/s40820-022-00824-6.

## Highlights

- A redox couple is employed to fulfill the dual-function of both heat-to-electricity conversion and ionic crosslinking.
- High thermoelectrochemical performance can be achieved at optimized redox concentration, and dual-crosslinked network ensures remarkable mechanical flexibility and robustness.
- The hydrogel-based quasi-solid-state thermocell can provide stable energy output under harsh mechanical stress and deformations.

**Supplementary Information:**

The online version contains supplementary material available at 10.1007/s40820-022-00824-6.

## Graphical abstract

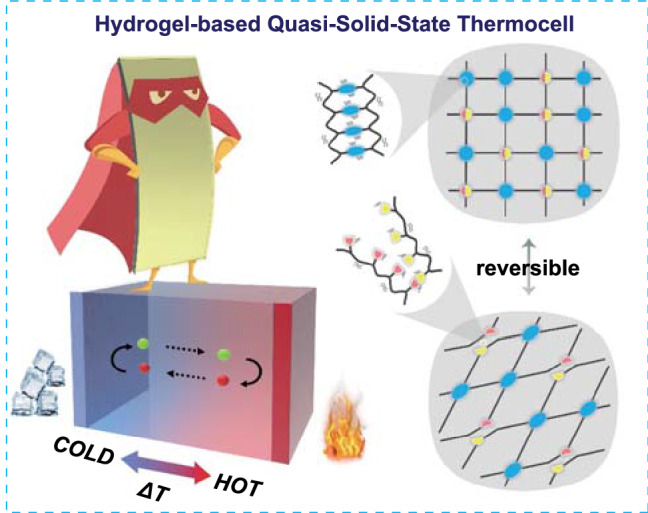

## Introduction

The booming of wearable electronics has stimulated the urgent demand for flexible, robust and efficient energy supply systems [1–3]. Compared to conventional energy techniques such as supercapacitors, batteries and solar cells [4–7], the direct utilization of body heat provides an attractive solution for energy supply, as heat is continually emitted from human body (up to 20 mW cm^−2^), so that it can serve as a constant power source [8, 9]. Driven by temperature gradient, thermoelectric devices can direct convert thermal energy into electricity, and they do not produce any audible noise or waste emission. The performance of thermoelectric materials is evaluated by the figure of merit *ZT*, which is further expressed by the Seebeck coefficient (*S*_*e*_), electrical conductivity (*σ*) and thermal conductivity (*κ*) (*ZT* = *S*_*e*_^2^*σT*/*κ*) [10]. Traditional solid thermoelectric materials have been extensively studied in the past decades. Although they have demonstrated good energy conversion efficiency at high temperatures, their application in low-grade heat harvesting is substantially hindered by the relatively low Seebeck coefficient near room temperature (on the order of µV K^−1^) [11–18], not to mention that their mechanical rigidity, high cost and toxicity would further hamper their development in flexible and wearable applications [19–22]. Alternatively, thermoelectrochemical cells (or thermocells, TECs) offer a flexible, cost-efficient and scalable route for direct heat-to-electricity conversion. TECs realize energy conversion via thermogalvanic effect, and they typically possess a much higher *S*_*e*_ (on the order of mV K^−1^) compared to traditional solid thermoelectric materials [23–26]. Considering the limited temperature difference between heat sources (*e.g.*, body heat) and the ambient, the development of TECs with a high *S*_*e*_ near room temperature is of great significance, because it can potentially increase the output voltage under a small temperature gradient.

A TEC comprises two electrodes and a redox couple-containing electrolyte. The equilibrium of the reversible redox reactions near the electrodes would be broken once a temperature gradient is built between the two electrodes, thus causing a potential difference [26–28]. To achieve higher energy conversion efficiency, efforts have been devoted to the optimization of electrode materials and the regulation of redox ions and electrolyte solvents [29–33]. Whereas, for wearable applications, conventional liquid-based electrolytes would bring about complicated packaging and integration issues. To circumvent these issues, one possible route is to solidify liquid electrolytes into quasi-solid-state hydrogel electrolytes. Some recent studies have demonstrated the successful utilization of hydrogel electrolytes to enable flexible TECs [34–39]. Hydrogel electrolytes are typically solid swollen polymer networks absorbing large amounts of aqueous solution, and they usually possess decent ion-conducting capability without the risk of liquid leakage [40, 41]. Besides, their mechanical softness is a highly desirable feature in fabricating flexible TECs.

Nevertheless, conventional hydrogel electrolytes prepared via simply mixing polymer aqueous solution with electrolyte salt [37, 42], or via the polymerization of one single monomer [41, 43, 44], are usually poor in mechanical strength, which can hardly provide consistent thermoelectrochemical performance under practical conditions, as they would be easily broken or damaged when under severe external mechanical stress (such as intense shearing force, high pressure and sharp cut). Moreover, they are usually very notch-sensitive, and once a notch is induced, their mechanical strength would drastically decay, leading to the inevitable fracture [45]. Thus, these reported hydrogel electrolytes can only serve for the purpose of flexibility, while the concern of mechanical robustness and toughness remains unattended. By introducing effective energy dissipation mechanisms, hydrogels can be made much more robust and notch-insensitive. For example, through the sacrificing of covalent bonds, double-network hydrogels consisting of two interpenetrating polymer networks can realize efficient energy dissipation [46]. While the major drawback of constructing such a sacrificial network is that, the rupture of covalent bonds would leave permanent damage to the hydrogels, and the original mechanical strength is thereby lost.

Herein, we utilize Fe^3+^/Fe^2+^ redox couple to synergistically regulate the thermoelectrochemical performance and mechanical robustness of flexible quasi-solid-state TECs. The temperature-dependent redox reactions of Fe^3+^/Fe^2+^ endow the hydrogel electrolyte with excellent heat-to-electricity conversion capability, and the ionic crosslinking based on the multivalent Fe^3+^/Fe^2+^ ions give rise to a sacrificial yet recoverable network for effective energy dissipation. At the optimized Fe^3+^/Fe^2+^ concentration, the hydrogel electrolyte demonstrated a high *S*_*e*_ of 1.43 mV K^−1^, and exhibited an impressive fracture toughness of 3555 J m^−2^, thus offering stable thermoelectrochemical performance against various harsh mechanical stimuli. The flexible quasi-solid-state TEC based on the mechanically robust hydrogel electrolyte offers a promising route to promote the development of wearable electronics.

## Experimental Section

### Materials and Preparation of Hydrogels

Acrylamide (≥ 99.0%, Aladdin), *N, N′*-methylenebisacrylamide (MBAA, Macklin), ammonium persulphate (≥ 99.0%, Macklin), sodium alginate (Aladdin), FeCl_3_ (Macklin), FeCl_2_·4H_2_O (Macklin) were purchased and used as received. The pristine PAAm was prepared as below: 8.722 g acrylamide monomer was dissolved in 40 mL deionized water to form a clear solution (21.8 wt% AAm compared to water). 5.25 mg MBAA was added as covalent crosslinker (0.06 wt% crosslinker compared to AAm), and 42.9 mg ammonium persulfate was added as initiator, followed by 0.5 h tiring at room temperature. The precursor solution was degassed by ultrasonic treatment, and the resultant homogenous solution was poured into glass moulds and placed in 65 °C oven overnight for polymerization. For the preparation of PAAM/Alg, 1.077 g sodium alginate was added and dissolved before polymerization (12.3 wt% alginate compared to AAm, resulting in the ion exchange capacity of 76.45 mmol L^−1^ for Fe^2+^ and 50.89 mmol L^−1^ for Fe^3+^, respectively). For the preparation of PAAM/Fe-Alg, the obtained hydrogels after polymerization were further immersed in Fe^3+^/Fe^2+^ aqueous solution with difference concentrations for ion exchange under a certain time. The TECs with doubled AAm content and doubled MBAA content were prepared in the same procedure with the addition of 43.6 wt% AAm monomer (compared to water) and 0.12 wt% crosslinker (compared to AAm), respectively.

### Morphological and Structural Characterizations

The micro morphology observations of the hydrogels were conducted on a thermo APREO S(A5-112) high resolution scanning electron microscope (SEM) with an acceleration voltage of 5 kV. FTIR spectra were obtained via a Fourier transform infrared spectroscopy (ATR-FTIR, PerkinElmer Spectrum 3) scanned in the wavenumber range of 4000–450 cm^−1^ with 64 scans and a nominal resolution of 4 cm^−1^ at room temperature. The surface chemical composition of the hydrogels was analyzed via a Thermo ESCALAB 250XI X-ray photoelectron spectroscopy.

### Mechanical Property Tests

The tensile stress–strain tests and hysteresis loops were performed using a tensile tester (SANS EUT4103) with a 50 N load at a rate of 50 mm min^−1^ in the air at room temperature. The thickness, length and width of the hydrogel samples were set to 2, 50, and 10 mm, respectively, and the gauge length was set to 25 mm. The fracture energy was determined by employing the approach proposed by Rivlin et al*.* [47]. Specifically, two samples (15.0 mm wide, 2.0 mm thick) of the same hydrogel were pulled by the tensile tester, and the distance between the two clamps was 20 mm. Before testing, one sample was intact without notch, and the other was half-notched in width (the precise length of notch is not important for this measurement). Force-distance curves were subsequently obtained via stretching these two hydrogels at a rate of 50 mm min^−1^, and the area under the curve represents the work done by the applied force, *W*(*L*) (as illustrated in Fig. S1). Upon the notch turns into a running crack, the fracture energy can be accordingly calculated from Eq. 1:

1 $$\Gamma = \frac{{W\left( {L_{c} } \right)}}{{a_{0} \times b_{0} }}$$

where *W*(*L*_c_) is the area under the force–length curve of unnotched sample at the critical distance *L*_c_, where the notch turns into a running crack in the notched sample, *a*_0_ and *b*_0_ are the original width and thickness of samples, respectively.

### Thermoelectrochemical Performance Measurements

The *S*_*e*_ of the TECs was determined by the fitting slope of the curve of the thermoelectrochemical potential Δ*V* versus the temperature difference Δ*T*, i.e., *S* =  − Δ*V*/Δ*T*. The Δ*V* − Δ*T* curve was obtained on a self-established apparatus, as shown in Fig. S2a. Specifically, the *S*_*e*_ at room temperature was measured by keeping the cold end of the TEC at around 25 °C, while the hot end was elevated to a series of higher temperatures starting from 25 °C at a fixed interval of 2 °C. The *S*_*e*_ at 37 and 50 °C was measured in a similar procedure except that the cold end was kept at 37 and 50 °C, respectively. During measurement, the two ends of the TEC were sandwiched between two big copper plates, whose temperature was manipulated by ohmic heaters. Two thermocouples were attached to the two ends of the TEC, respectively, and a temperature controller was employed to accurately control the temperature of the hot end and cold end. Copper wires were drawn from the two ends of the TEC and connected to a Keithley 2700 data acquisition, so that the data of voltage *V* and current *I* can be collected. The output power *P* was calculated according to Joule’s law (*P* = *IV*). The ionic conductivity of the TECs was measured by using an electrochemical workstation (Chenhua 660e). Specifically, the TECs were kept at different temperatures (25, 37, and 50 °C) or under different stretching strains (Fig. S2b), and the corresponding Nyquist plots were obtained by electrochemical impedance spectroscopy (EIS). The resultant ionic conductivity σ was calculated according to the following equation *σ* = *L*/(*R*_*b*_ × *A*), where *L* (cm) is the distance between the two electrodes, *R*_*b*_ (Ω) is the bulk resistance (intercept at Z’ axis), and *A* (cm^2^) is the contact area between the sample and the electrodes. Regarding the thermoelectrochemical stability against harsh mechanical stimuli, the TECs were fitted with electrodes on the extremities and padded with polyimide tape to avoid short circuit, and the output voltage was recorded on the self-established apparatus after various operations including bending, twisting, hammering and cutting. At least five data values were measured for each operation and the average value was adopted.

## Results and Discussion

### Forming of Hydrogel-Based TECs

**Fig. 1:**
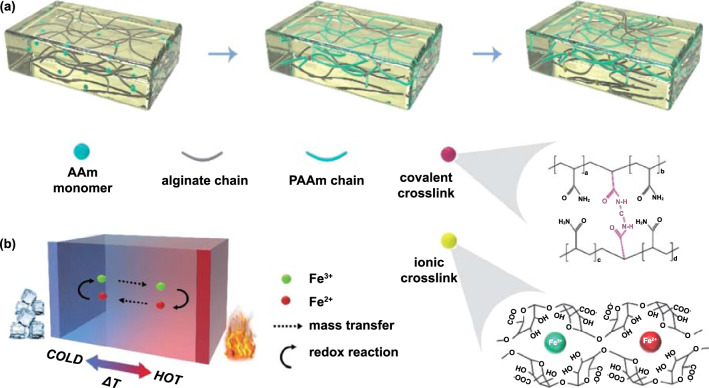
**a** Illustration of the forming process of the covalently-crosslinked network and the ionically-crosslinked network within the hydrogel body. The molecular schematics reveal the structures of covalent and ionic crosslinks. **b** Illustration of the working mechanism of a TEC based on thermogalvanic effect

The hydrogel used as electrolyte for quasi-solid-state TECs was constructed with two crosslinking networks, *i.e.*, covalently-crosslinked polyacrylamide (PAAm) network and ionically-crosslinked alginate network, as illustrated in Fig. 1a. The detailed preparation process is depicted in Fig. S3, which involves the building of PAAm covalent network, and an ion exchange step that forms an ionic crosslinking network. In this design, Fe^3+^/Fe^2+^ redox couple was utilized not only as ionic crosslinkers, but also accounted for the thermogalvanic effect. The two crosslinking structures are presented in the lower right corner of Fig. 1a, and the heat-to-electricity conversion mechanism based on thermogalvanic effect is schematically shown in Fig. 1b.

### Characterizations of the Hydrogel

**Fig. 2:**
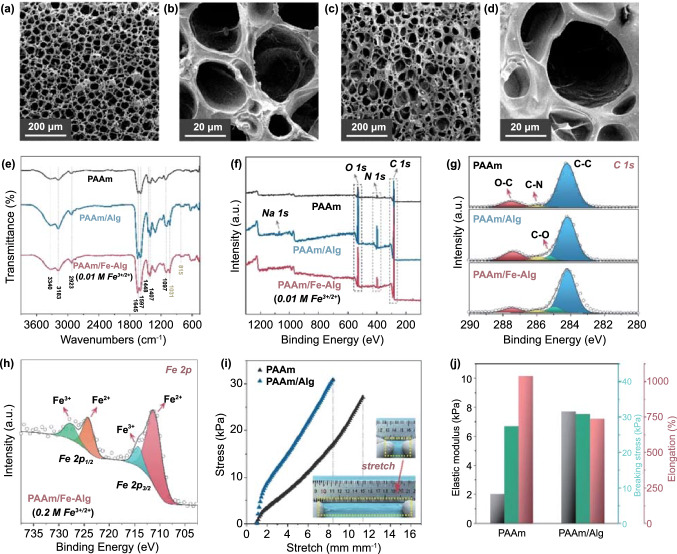
**a, b** SEM images of the porous structure of the PAAm hydrogel. **c, d** SEM images of the porous structure of the PAAm/Alg hydrogel. **e** FTIR spectra of various hydrogel samples. **f** XPS full spectra of various hydrogel samples. **g** XPS C 1* s* spectra of various hydrogel samples. **h** XPS Fe 2*p* spectrum of the PAAm/Fe-Alg hydrogel ion-exchanged with 0.2 M Fe^3+^/Fe^2+^ solution. **i** Stress–strain curves of the PAAm and PAAm/Alg hydrogels. Inset shows the stretching process of the PAAm/Alg hydrogel, which can be easily stretched over 700%. **j** The elastic modulus, breaking stress and elongation data of the PAAm and PAAm/Alg hydrogels

For simplification, the PAAm hydrogel with the presence of alginate before ionic crosslinking is denoted as PAAm/Alg, and the hydrogel after ionic crosslinking is denoted as PAAm/Fe-Alg. As revealed by SEM, the pristine PAAm hydrogel exhibited an interconnected porous structure with a pore size distribution of 10–30 µm (Fig. 2a and b). After the introducing of alginate, the PAAm/Alg hydrogel retained a similar porous structure (Figs. 2c and d and S4). The porous structure is crucial for the hydrogels to function as solid-state electrolytes, as it provides facile pathways for ion transport. The incorporation of alginate slightly increased the pore size of the hydrogel, as revealed in Fig. S5, which may induce change in mechanical properties. The structural information of the hydrogels was verified by FTIR and X-ray photoelectron spectroscopy (XPS). As shown in Fig. 2e, the FTIR spectra indicate that PAAm and alginate molecules retained their original structures, suggesting the physical interactions of the two kinds of molecular chains. The bands at around 3340 and 3183 cm^−1^ (N–H stretching vibration), 2923 cm^−1^ (*sp*^3^ C–H bonds), 1645 and 1597 cm^−1^ (amide I and II), 1448 cm^−1^ (CH_2_ in-plane scissoring), 1407 cm^−1^ (primary amide C–N stretching), and 1097 cm^−1^ (C–O stretching in CH–OH) were detected for all samples [48, 49]. After adding alginate, a new peak at 1031 cm^−1^ emerged, which belongs to the symmetric C–O stretching [50]. As revealed by the XPS spectra (Figs. 2f and h and S6–S7), besides C 1* s*, N 1* s* and O 1* s*, Na 1* s* and Fe 2*p* binding energies were also detected for PAAm/Alg and PAAm/Fe-Alg, indicating the presence of alginate and iron (Fe signal was not detected in the sample with low Fe^3+^/Fe^2+^ concentration, but detected in the sample with higher Fe^3+^/Fe^2+^ concentration, as shown in Fig. S6). The mechanical properties were also preliminarily evaluated for the pristine PAAm and PAAm/Alg (subject to Fe^3+^/Fe^2+^ concentration, the mechanical properties of PAAm/Fe-Alg will be discussed later). As shown in Fig. 2i, the pristine PAAm can be stretched over 1100%. With the introduction of alginate, the stretchability was slightly affected, but the sample can still be stretched over 700%. Figure S8 and the inset of Fig. 2i visually demonstrate the highly stretchable feature of the hydrogels. The data of elastic modulus, breaking stress and elongation at break for PAAm and PAAm/Alg were listed in Fig. 2j. In general, compared to the pristine PAAm, the incorporation of alginate led to moderately decreased stretchability but slightly increased breaking stress and notably improved elastic modulus.

### Thermoelectrochemical Performance of the TECs

**Fig. 3:**
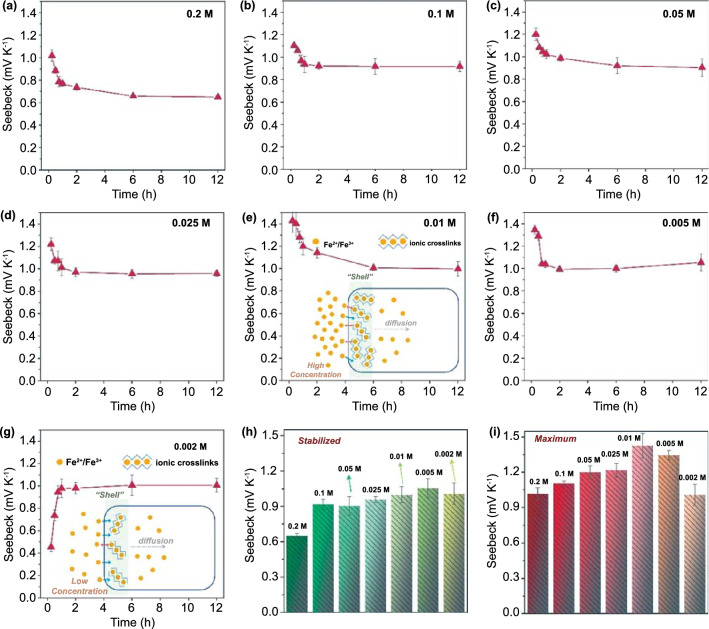
The Seebeck coefficient as a function of ion exchange time of different PAAm/Fe-Alg hydrogels ion-exchanged with **a** 0.2 M, **b** 0.1 M, **c** 0.05 M, **d** 0.025 M, **e** 0.01 M, **f** 0.005 M and **g** 0.002 M Fe^3+^/Fe^2+^ aqueous solutions. Insets of **e** and **g** are the illustrations showing the ion diffusion process during ion exchange. Higher redox couple concentration would result in higher “shell” concentration (more crosslinks in “shell” zone), and the ion diffusion into the inner area of the hydrogel would be more hindered (inset of **e**); Lower redox couple concentration could result in lower “shell” concentration, and the subsequent ion diffusion into the inner area of the hydrogel could be easier (inset of **g**). **h** Comparison of the stabilized *S*_*e*_ of different PAAm/Fe-Alg hydrogels (the *S*_*e*_ at the ion exchange time of 6 h). **i** Comparison of the maximum *S*_*e*_ of different PAAm/Fe-Alg hydrogels (the *S*_*e*_ at 0.25 h for the 0.2, 0.1, 0.05, 0.025, 0.01, and 0.005 M samples, and the *S*_*e*_ at 6 h for the 0.002 M sample)

The PAAm/Fe-Alg hydrogel electrolyte was obtained through the ion exchange between the PAAm/Alg hydrogel and the Fe^3+^/Fe^2+^ solution. In the PAAm/Fe-Alg, the temperature-dependent redox reactions between Fe^3+^ and Fe^2+^ ions can induce a potential difference between the hot end and cold end of the electrolyte. The generated potential difference, *i.e.*, thermovoltage, is characterized by the thermoelectrochemical Seebeck coefficient *S*_*e*_. It has been agreed that the concentration of redox couple plays an important role in deciding energy conversion efficiency [23, 51]. Therefore, the effect of Fe^3+^/Fe^2+^ concentration on the *S*_*e*_ of the PAAm/Fe-Alg hydrogel electrolyte was thoroughly investigated. The measured *S*_*e*_ values for the hydrogel electrolytes ion-exchanged with different Fe^3+^/Fe^2+^ concentrations (0.2 ~ − 0.002 M) are presented in Fig. 3a–g. Note that the actual Fe^3+^/Fe^2+^ redox couple concentration within the hydrogel electrolytes should be co-determined by the concentration of solutions and the ion exchange time.

It can be seen that most hydrogel electrolytes exhibited decreased *S*_*e*_ with increasing ion exchange time, and a plateau value was reached at the ion exchange time of 6 h. While for the 0.002 M sample, the *S*_*e*_ increased with increasing ion exchange time until a plateau was reached at 6 h. That is to say, an equilibrium concentration was built within the hydrogel electrolytes after 6 h of ion exchange. The stabilized *S*_*e*_ values for various samples are compared in Fig. 3h, which reveals a general trend of gradual increase with decreasing concentration until 0.005 M, and further decreasing concentration to 0.002 M led to slightly reduced *S*_*e*_. Considering the well-established equation *S*_*e*_ = *∂V/∂T* = Δ*S*/*nF* (where Δ*S* is the entropy difference of redox reactions, *n* is the number of transferred electrons, and *F* is the Faraday’s constant) [26, 27], it is claimed that more water molecules are capable to independently solvate the redox ions at decreased ion concentrations, thereby altering the difference in solvation environment, resulting in enlarged difference in reaction entropy [16]. While the solvent reorientation cannot well maintain under further decreased ion concentrations, which is possibly due to a subtle balance of self-reorientation dynamics *vs.* solute-imposed reorientation [52]. Therefore, in this case, *S*_*e*_ increased with decreasing Fe^3+^/Fe^2+^ concentration and peaked at a relatively low concentration. Besides, the maximum *S*_*e*_ values for various samples (the *S*_*e*_ at 0.25 h for the 0.2, 0.1, 0.05, 0.025, 0.01, and 0.005 M samples, and the *S*_*e*_ at 6 h for the 0.002 M sample) are compared in Fig. 3i.

The notable change in *S*_*e*_ under different ion exchange time periods should be attributed to the complicated diffusion dynamics during ion exchange, which is dominated by both the solution concentration (external ion concentration) and the dual-crosslinked networks of the hydrogels. As a result, the actual redox couple concentration within the hydrogels is a function of both external concentration and ion exchange time. According to the results shown in Fig. 3, it is generally assumed that ion exchange was completed when the ion exchange time reached 6 h. Thus, the optimal solution concentration for ion exchange regarding the *S*_*e*_ should locate at around 0.01 ~ 0.002 M (Fig. 3h). Whereas, this cannot account for the sharp difference between Fig. 3a–f and Fig. 3g. In the former figures, the *S*_*e*_ showed a decrease trend with the increase of ion exchange time, while in the latter an opposite trend is observed. Considering the multivalent iron ions of the redox couple, the ionic crosslinks should severely affect the ion exchange process, *i.e.*, the diffusion kinetics. As illustrated in the insets of Fig. 3e–g, ionic crosslinks would immediately form in the outer area of the hydrogel during ion exchange because of the direct contact with the multivalent Fe^2+^ and Fe^3+^ ions, thus forming a relatively rigid “shell”. Due to concentration gradient-driven diffusion and electrostatic repulsive force, this rigid “shell” would in return disturb the subsequent ion diffusion process, resulting in the uneven distribution of redox ions within the hydrogel body. With higher redox ion concentration, as in the case of the inset of Fig. 3e, such “shell effect” was more obvious, thus substantially affecting the subsequent ion diffusion. With lower redox ion concentration, as in the case of the inset of Fig. 3g, such “shell effect” was less notable. Results indicate that the PAAm/Fe-Alg ion-exchanged with 0.01 M Fe^3+^/Fe^2+^ for 0.25 h possessed the optimum “actual concentration” and showed the highest *S*_*e*_ of 1.43 mV K^−1^, although the solution concentration of 0.01 M doesn’t really represent the actual ion concentration within the hydrogel. For Fig. 3a–f, longer ion exchange time led to higher actual ion concentration within the hydrogel, and the corresponding *S*_*e*_ accordingly decreased. For Fig. 3g, longer ion exchange time also led to higher overall ion concentration within the hydrogel, while the corresponding *S*_*e*_ increased instead. This phenomenon indicates that, the distribution of redox ions within the hydrogel has a gradient, and the optimal overall concentration should locate between 0.002 and 0.005 M, despite the fact that the optimal ion exchange option fell at 0.01 M + 15 min. It is also noticed that the *S*_*e*_ of the PAAm/Fe-Alg hydrogel electrolytes is positive, and its value matches well with the widely-studied 0.4 M Fe(CN)_6_^3−^/Fe(CN)_6_^4−^ aqueous electrolyte (*S*_*e*_ =  − 1.4 mV K^−1^), suggesting the high potential to assemble p-n connections for higher energy output [53].

**Fig. 4:**
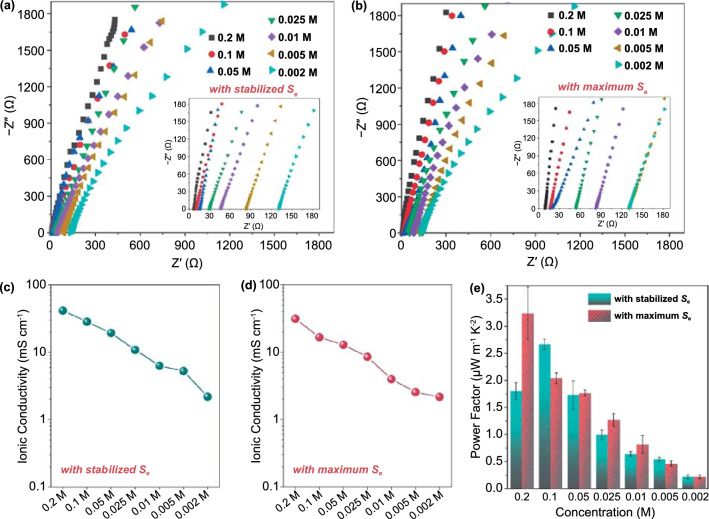
**a** EIS spectra of various PAAm/Fe-Alg hydrogels with stabilized *S*_*e*_. **b** EIS spectra of various PAAm/Fe-Alg hydrogels with maximum *S*_*e*_. **c** Ionic conductivity of various PAAm/Fe-Alg hydrogels with stabilized *S*_*e*_. **d** Ionic conductivity of various PAAm/Fe-Alg hydrogels with maximum *S*_*e*_. **e** The calculated power factors of various PAAm/Fe-Alg hydrogels

As revealed by the SEM images in Fig. 1, the porous structure of the hydrogels theoretically allows for ions to freely transport, which is essential for the continuous operation of TECs. During the thermoelectrochemical process, oxidation occurs at cathode with electrons transferred from electrolyte to the cathode, and reduction occurs at anode with electrons retrieved from the anode to electrolyte. As a result, a concentration gradient of redox ions is built across the electrolyte, thus driving the transport of ions throughout the electrolyte. Therefore, ion transport directly relates to the redox reaction rate and hence the heat-to-electricity conversion efficiency [31]. To investigate the ion transport, the ionic conductivity (*σ*) of the PAAm/Fe-Alg hydrogel electrolytes was studied by EIS. As revealed in Fig. 4a, c, the hydrogel electrolytes with stabilized *S*_*e*_ exhibited decreased *σ* with the decrease of ion exchange concentration. Figure 4b, d indicates the same trend for the hydrogel electrolytes with maximum *S*_*e*_. Since prolonging ion exchange time resulted in higher actual concentration within hydrogels, the hydrogel electrolytes with stabilized *S*_*e*_ generally possessed higher *σ* than that of the hydrogel electrolytes with maximum *S*_*e*_. Specifically, the 0.2 M-6 h PAAm/Fe-Alg showed the highest *σ* of 41.6 mS cm^−1^, and the 0.01 M-0.25 h PAAm/Fe-Alg showed a much lower *σ* of 4.0 mS cm^−1^ due to the low ion exchange concentration. The corresponding power factors (*S*_*e*_^*2*^*σ*) for various samples are presented in Fig. 4e. The *σ* as well as the *S*_*e*_ for the samples with varied monomer content and crosslinker content was also investigated and the results are shown in Fig. S9.

### Mechanical Properties of the TECs

**Fig. 5:**
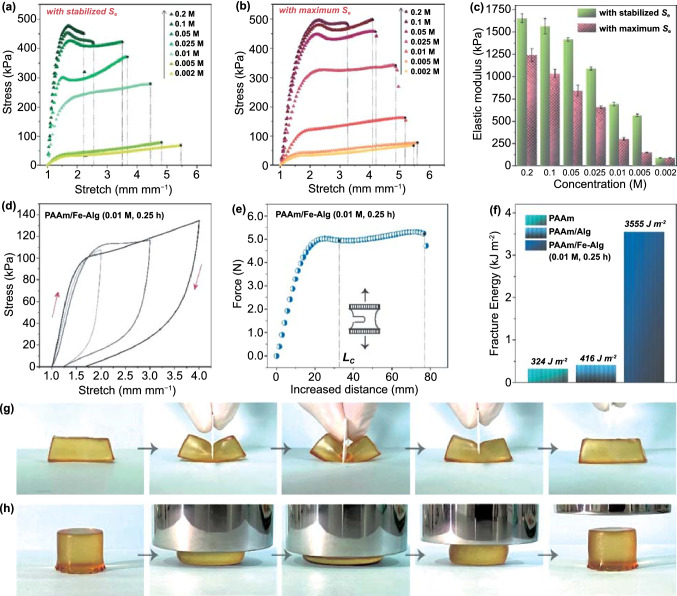
**a** Stress–strain curves of various PAAm/Fe-Alg hydrogels with stabilized *S*_*e*_. **b** Stress–strain curves of various PAAm/Fe-Alg hydrogels with maximum *S*_*e*_. **c** Elastic modulus of various PAAm/Fe-Alg hydrogels. **d** Hysteresis loops of the 0.01 M-0.25 h PAAm/Fe-Alg hydrogel. **e** Force-distance curve of the 0.01 M-0.25 h PAAm/Fe-Alg hydrogel for the calculation of facture toughness. L_c_ refers to the critical distance when the notch turned into a running crack. **f** Facture toughness of the pristine PAAm, PAAm/Alg and 0.01 M-0.25 h PAAm/Fe-Alg hydrogels. **g** Photos showing the cutting process performed on the 0.01 M-0.25 h PAAm/Fe-Alg hydrogel. **h** Photos showing the compressing process performed on the 0.01 M-0.25 h PAAm/Fe-Alg hydrogel

The Fe^3+^/Fe^2+^ redox ions not only endow the hydrogel with thermogalvanic effect, but also serve as ionic crosslinkers to form a second network. Therefore, the Fe^3+^/Fe^2+^ concentration would substantially affect the mechanical properties of the PAAm/Fe-Alg hydrogel electrolyte. According to the stress–strain curves presented in Fig. 5a and b, for both the hydrogel electrolytes with stabilized *S*_*e*_ and the hydrogel electrolytes with maximum *S*_*e*_, their mechanical strength rapidly increased with the increase of Fe^3+^/Fe^2+^ concentration, suggesting the effective ionic crosslinking with alginate chains. It is noticeable that the stress–strain behavior of the PAAm/Fe-Alg samples (≥ 0.01 M) significantly differ from that of the pristine PAAm and PAAm/Alg (Fig. 2i): the pristine PAAm and PAAm/Alg showed simple elastic behavior upon stretching, while the PAAm/Fe-Alg samples (≥ 0.01 M) exhibited a two-stage stress–strain behavior that separated by a yield point. This two-stage stress–strain behavior should be attributed to the dual-crosslinked structure of the hydrogels, where two crosslinked networks synergistically deform during the tensile test [54]. As shown in Fig. 5c, the highest elastic modulus of 1654.7 kPa was achieved for the hydrogel electrolyte with the highest ion concentration, *i.e.*, the 0.2 M-6 h PAAm/Fe-Alg hydrogel electrolyte. This value is significantly larger than that of the pristine PAAm and PAAm/Alg hydrogels (which are less than 10 kPa). The breaking stress exhibited a similar trend as elastic modulus (Fig. S10), while the stretchability drastically decreased upon the incorporation of Fe^3+^/Fe^2+^ ions (Fig. S11), suggesting the gain in rigidity and the loss in flexibility. For wearable applications, high thermoelectrochemical performance, high mechanical strength and high flexibility are all desired, while the results show that the three merits can hardly be simultaneously optimized. Thus, a trade-off must be considered. As shown in Fig. 5a and b, a distinct increase in mechanical strength can be observed for the 0.01 M PAAm/Fe-Alg compared to the PAAm/Alg. Besides, as discussed previously, the 0.01 M-0.25 h PAAm/Fe-Alg exhibited the highest *S*_*e*_ among all samples, and it showed a moderate conductivity. Moreover, the 0.01 M-0.25 h PAAm/Fe-Alg can still be stretched over 400%, indicating a satisfactory mechanical flexibility. Its high stretchability was demonstrated in Fig. S12. Therefore, the 0.01 M-0.25 h PAAm/Fe-Alg offered a subtle balance among thermoelectrochemical performance, mechanical strength and flexibility (Fig. S13), and it was chosen as the hydrogel electrolyte for subsequent flexible TEC fabrication and evaluation.

Toughened by the ionically-crosslinked Fe-alginate network, the 0.01 M-0.25 h PAAm/Fe-Alg hydrogel electrolyte can dissipate energy much more effectively, as revealed by the hysteresis loops in Figs. 5d and S14. Hysteresis is a measurement of material toughness, and the area enclosed by the loops represents the energy dissipated during the loading/unloading cycle [46]. The hysteresis of pristine PAAm and PAAm/Alg is neglectable, while for the 0.01 M-0.25 h PAAm/Fe-Alg, the hysteresis is remarkable, and the dissipated energy reached 238.8 kJ m^−3^ when stretching to 300%. Such large hysteresis is mainly ascribed to the unzipping of the reversible ionic bonds, which are physical crosslinks and can be self-recovered upon the unloading of stress.

The reversible ionic bonds are also key to attaining high fracture toughness. Fracture toughness indicates the notch-insensitivity of hydrogels, thus it’s highly desired in terms of stability and durability in practical use. To determine the fracture toughness, the hydrogel was notched and then stretched on a tensile tester until notch propagation (Figs. 5e and S15). The detailed calculation method of fracture toughness is presented in the Experimental Section and illustrated in Fig. S1, and the obtained results are given in Fig. 5f. As revealed, the 0.01 M-0.25 h PAAm/Fe-Alg exhibited a facture toughness of 3555 J m^−2^, significantly outperformed the pristine PAAm and the PAAm/Alg. The notable improvement in fracture toughness can be interpreted with Lake-Thomas model [55, 56]: for a notched hydrogel, the network near the notch endures much more stress than other zones during stretching, resulting in a running crack. In the case of the pristine PAAm and the PAAm/Alg, chain rupture only occurs in a small localized zone adjacent to the crack, thus the facture energy is small. In the case of the 0.01 M-0.25 h PAAm/Fe-Alg, on one hand, the covalently-crosslinked PAAm chains resist the crack and endure the deformation, on the other hand, the ionically-crosslinked alginate chains unzip over a large zone. In this way, the concentrated stress can be mitigated, and the energy can be dissipated to a large area, leading to a high fracture toughness (Fig. S16). Figure 5g and h demonstrates the excellent robustness of the 0.01 M-0.25 h PAAm/Fe-Alg hydrogel electrolyte, suggesting the effective energy dissipation. The hydrogel electrolyte was deeply cut through from top to bottom with a sharp blade, and then restored fully to its initial state with no visible damage left (Fig. S17). It was also severely compressed to one fourth of its original height and then fully recovered. Figure S18 further reveals its superior stretchability and strength by using it to hang a 500-g weight.

### Flexible and Tough TECs with Stable Energy Output

**Fig. 6:**
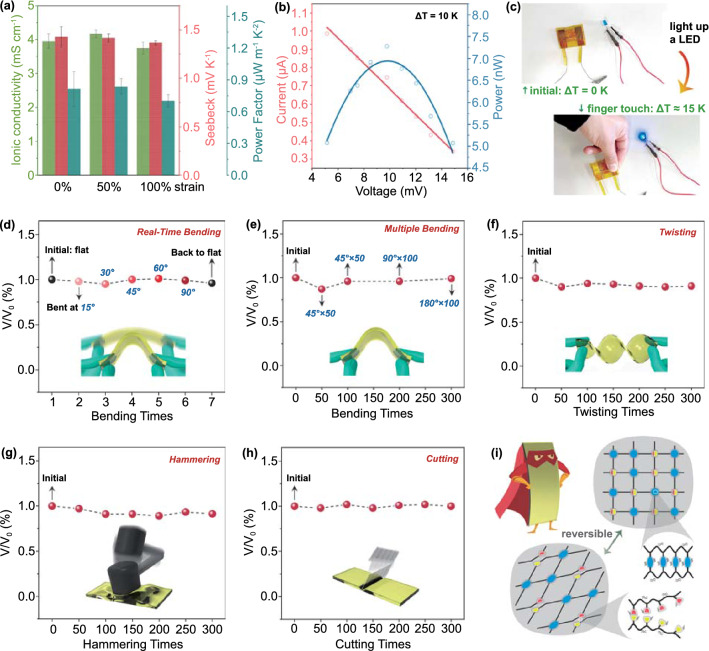
**a** Thermoelectrochemical performance of the TEC under various stretching strains. **b** Power output of single TEC under the temperature difference of 10 K. **c** Demonstration of lighting up a LED with a single thermocell under the temperature difference between the human body and the ambient. A voltage amplifier was used during the demonstration. **d** Output voltage fluctuation of the TEC after on-site bending at various angles. **e** Output voltage fluctuation of the TEC after bending up to 300 times. **f** Output voltage fluctuation of the TEC after twisting up to 300 times. **g** Output voltage fluctuation of the TEC after hammering up to 300 times. **h** Output voltage fluctuation of the TEC after cutting up to 300 times. **i** Illustration showing the effective energy dissipation mechanism upon harsh mechanical stimuli. The blue spheres represent covalent crosslinks, and the half red-half yellow spheres represent ironical crosslinks that undergo reversible break and recovery

Combining the designed thermogalvanic effect with the remarkable mechanical robustness, a flexible quasi-solid-state TEC using the 0.01 M-0.25 h PAAm/Fe-Alg hydrogel as electrolyte is subsequently demonstrated. Figure S19 verifies that the TEC possesses relatively stable thermoelectrochemical performance and mechanical properties under 37 °C (body temperature) and 50 °C (high environmental temperature), indicating a stable energy output under some expected application scenarios. As shown in Fig. 6a, it can also sustain stable thermoelectrochemical performance under the stretching strain of 50% and 100%. The thermal conductivity of the hydrogel was estimated to be around 0.1 − 0.2 W m^−1^ K^−2^ [34], resulting in a room temperature *ZT* of 0.002 − 0.003. With one small piece of the hydrogel (width = 5 mm, length = 40 mm, thickness = 2 mm) working as electrolyte, a single TEC was readily assembled using platinum as electrodes. The single TEC was encapsulated with polyimide films and fitted with copper wires. It can generate a maximum power of 9.77 nW under the small temperature difference of 10 K (Fig. 6b). With a voltage amplifier, the single TEC has the potential to power small electronics under the limited temperature difference between the human body and the ambient. As shown in Figs. 6c and S20, the blue LED was easily lit up when human fingers touched one end of the TEC. The output power and energy can also be easily increased by connecting multiple TECs in series and by enlarging the applied temperature difference. Thus, some low energy-demanding electronics can be powered by the TECs via the utilization of low-grade heat. For example, the wireless sensor nodes only consumed a small power of 295 pW [57], and the novel sub-nW data transmitter only consumed an average power of 78 pW [58]. In our previous work, we also reported a self-powered strain sensor initiated by a gel-based TEC through the harvesting of human body heat [59].

To demonstrate its advantageous mechanical robustness, the TEC was subject to various harsh mechanical stimuli, and the generated thermovoltage under a given temperature difference was accordingly recorded. It was firstly bent at different angles (Fig. 6d), and then experienced multiple bending up to 300 times (Fig. 6e). It also underwent the large deformation of twisting for 300 times (Fig. 6f). Furthermore, to simulate the high mechanical impact and stress that may be encountered in real wearable applications, the TEC was heavily hammered and cut for hundreds of times (Fig. 6g and h). Remarkably, it can still maintain relatively constant output voltage, and can work continuously without malfunction, demonstrating impressive stability and durability. SEM observations were conducted to examine the porous structure of the hydrogel electrolytes after various mechanical stimuli. As shown in Fig. S21, the hydrogels still held porous structure without collapse, suggesting good structural stability. Figure 6i illustrates the effective dissipate energy via the reversible rupture of ionic bonds and the deformation of covalent network, which accounts for the superior mechanical robustness of the TEC. Therefore, the as-fabricated TEC not only caters for the needs of flexibility, but also meet the requirement of mechanical robustness for wearable applications.

## Conclusions

In this study, a flexible quasi-solid-state TEC with outstanding thermoelectrochemical performance and mechanical properties was constructed. The thermogalvanic effect and mechanical robustness were simultaneously regulated via the multivalent cations of a redox couple. As a result, the TEC exhibited a high *S*_*e*_ of 1.43 mV K^−1^ with a significantly improved fracture toughness of 3555 J m^−2^ at the optimal redox couple concentration. It was demonstrated to sustain large deformations, high mechanical impact and intense mechanical stress that simulate the practical conditions of wearable applications, while miantained stable output voltage under a temperature difference. The remarkable mechanical robustness of the TEC originates from the dual-crosslinked structure of the hydrogel electrolyte, where the covalently-crosslinked network stabilizes the deformation and the ionically-crosslinked network buffers the concentrated stress, thus realizing effective energy dissipation. The flexible quasi-solid-state TEC proposed in this work showed great potential in wearable applications that require not only flexibility but also stability and durability.

## Supplementary Information

Below is the link to the electronic supplementary material.

Supplementary file1 (PDF 1580 kb)
