# Supplementary material for: Regulating Thermogalvanic Effect and Mechanical Robustness via Redox Ions for Flexible Quasi-Solid-State Thermocells
Source: Nanomicro Lett. 2022 Mar 25;14:81. doi: 10.1007/s40820-022-00824-6 (PMC8956784; doi:10.1007/s40820-022-00824-6)
Supplement: Supplementary file 1 — Supplementary file1 (PDF 1580 kb) [file 40820_2022_824_MOESM1_ESM.pdf]

Supporting Information for

Regulating Thermogalvanic Effect and Mechanical Robustness via Redox Ions for Flexible Quasi-Solid-State Thermocells

Peng Peng[1], Jiaqian Zhou[1], Lirong Liang[1], Xuan Huang[1], Haicai Lv[1, 2], Zhuoxin Liu[1, *], and Guangming Chen[1, *]

[1]College of Materials Science and Engineering, Shenzhen University, Shenzhen 518055, People's Republic of China

[2]College of Physics and Optoelectronic Engineering, Shenzhen University, Shenzhen 518060, People's Republic of China

*Corresponding authors. Email: liuzhuoxin@szu.edu.cn (Zhuoxin Liu); chengm@szu.edu.cn (Guangming Chen)

Supplementary Figures

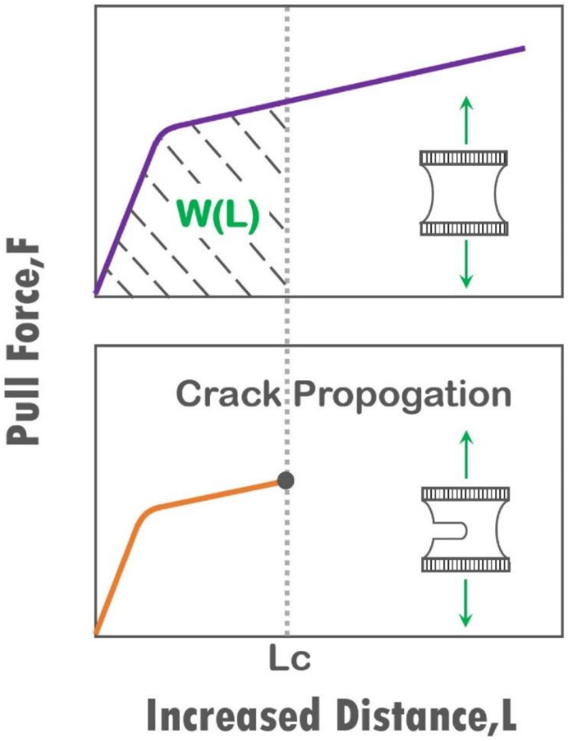

Fig. S1 Determination of facture toughness. The upper image illustrates the tensile test of the unnotched sample to obtain the force-length curve under which the area before L_c was calculated, the lower image illustrates the tensile test of the notched sample to determine the critical distance L_c when the notch turned into a running crack

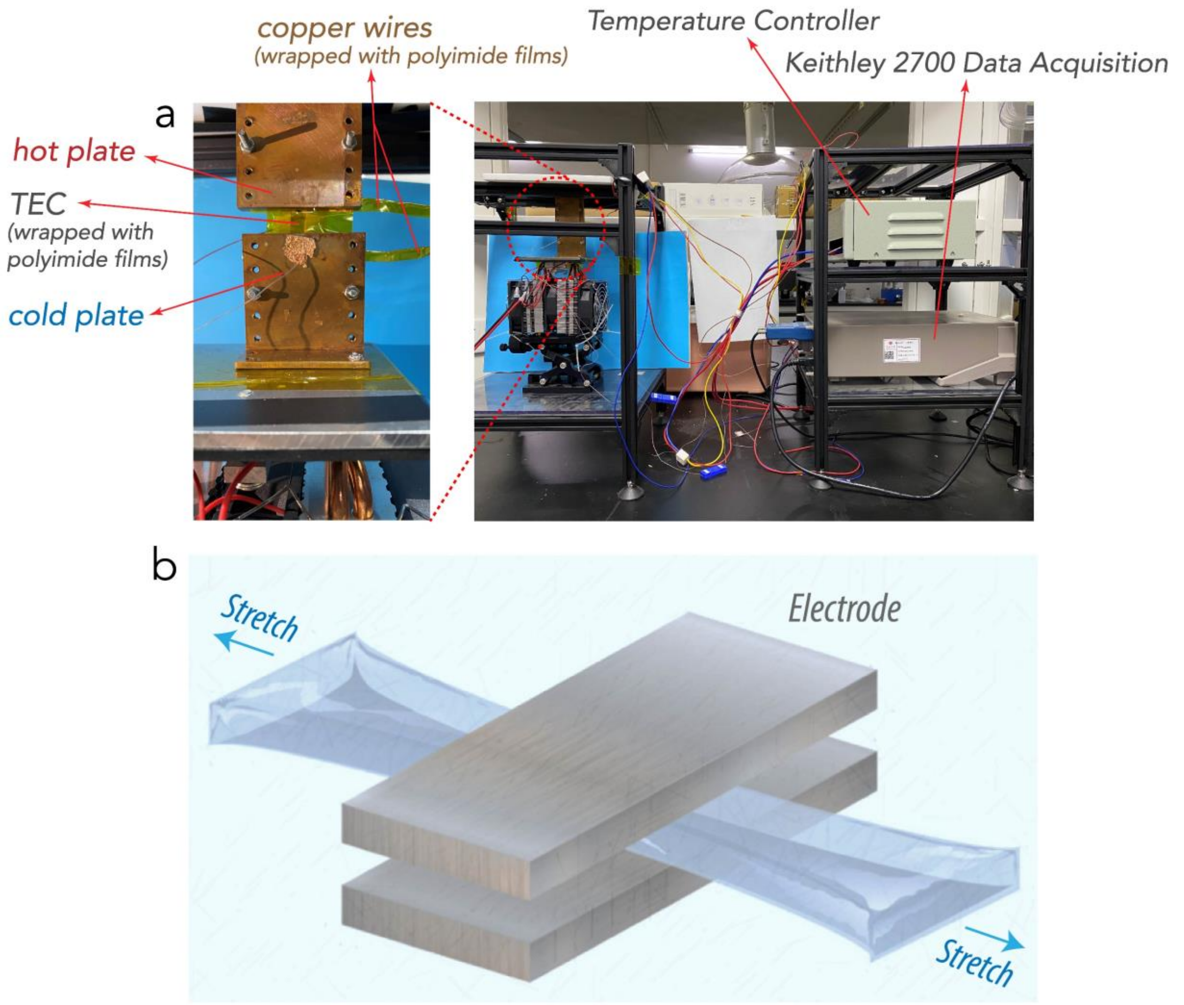

Fig. S2 a Photos showing the self-established apparatus for the measurement of the S_e and the device performance. **b** Illustration showing the measuring of ionic conductivity of the hydrogel under strains

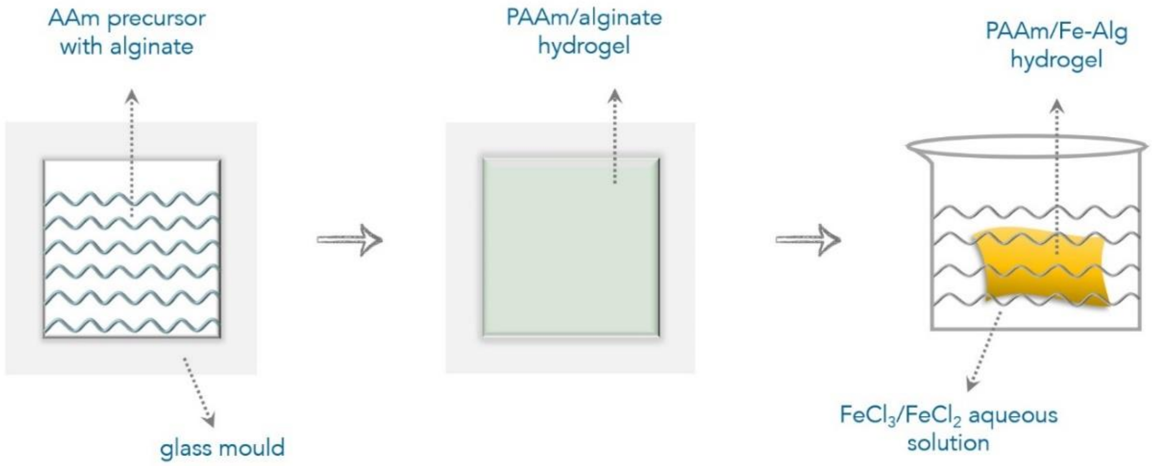

Fig. S3 Preparation process of the hydrogel electrolyte that involves the polymerization of PAAm and the ion exchange with Fe^{3+}/Fe^{2+} solution

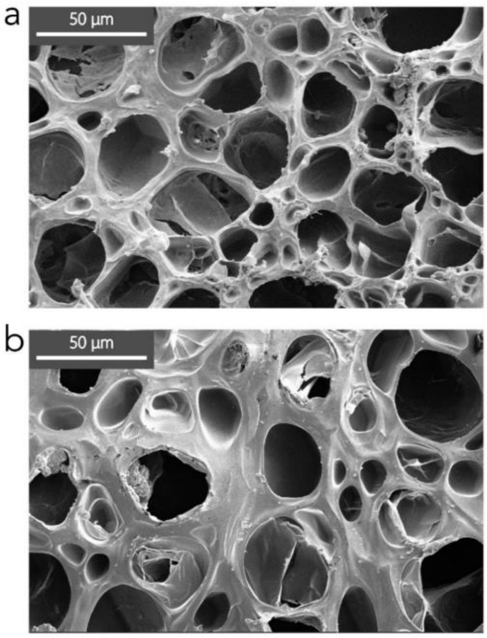

Fig. S4 SEM images of the **a** PAAm and **b** PAAm/Alg hydrogels at the magnification of 1000

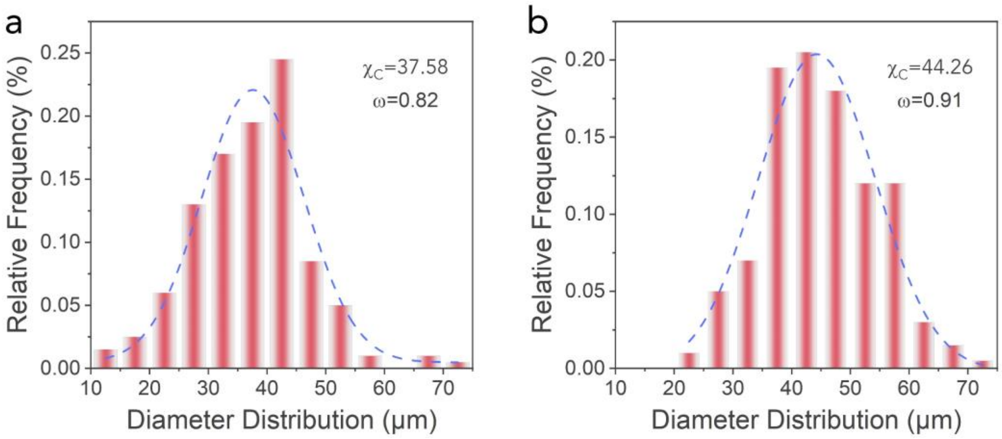

Fig. S5 Statistical analysis of the pore diameter distribution of **a** the pristine PAAm and **b** the PAAm/Alg. X_c refers to the average pore diameter value, and ω refers to the standard deviation

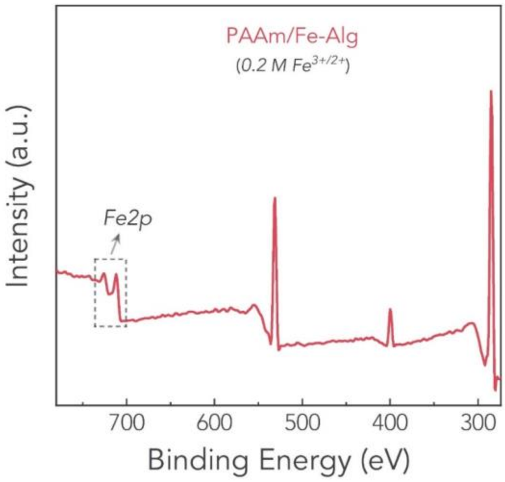

Fig. S6 XPS spectrum of the PAAm/Fe-Alg hydrogel ion-exchanged with 0.2 M Fe^{3+}/Fe^{2+} solution, where Fe2p signal was notably revealed

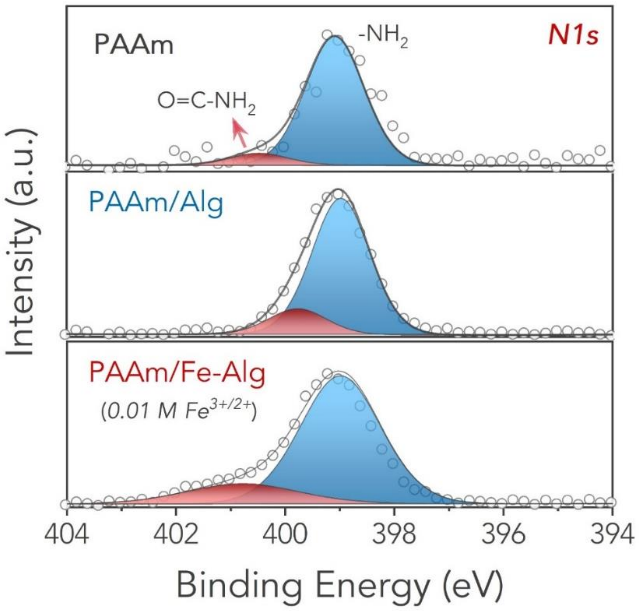

Fig. S7 XPS N 1s spectra of various hydrogel samples

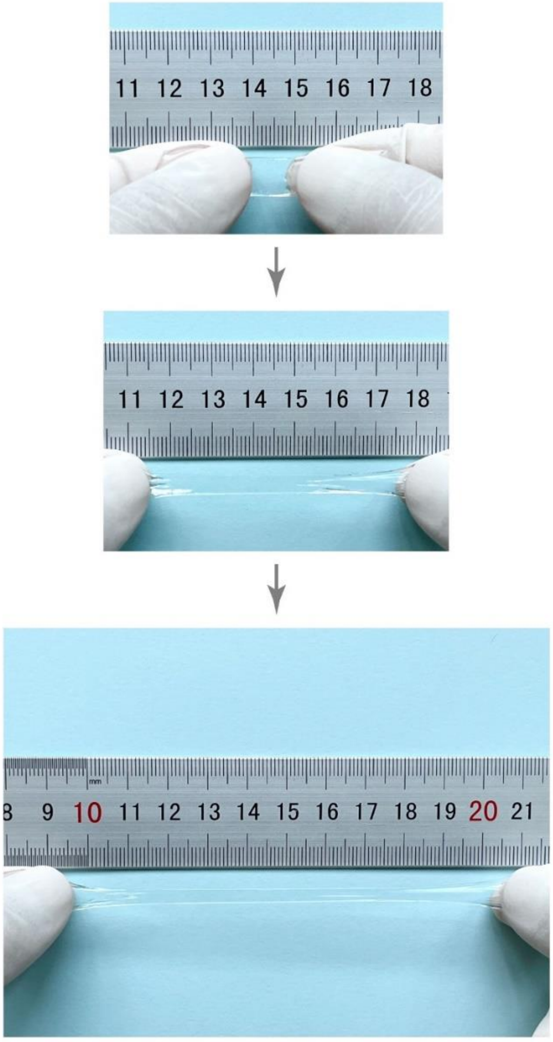

Fig. S8 Photos showing the stretching process of the PAAm hydrogel

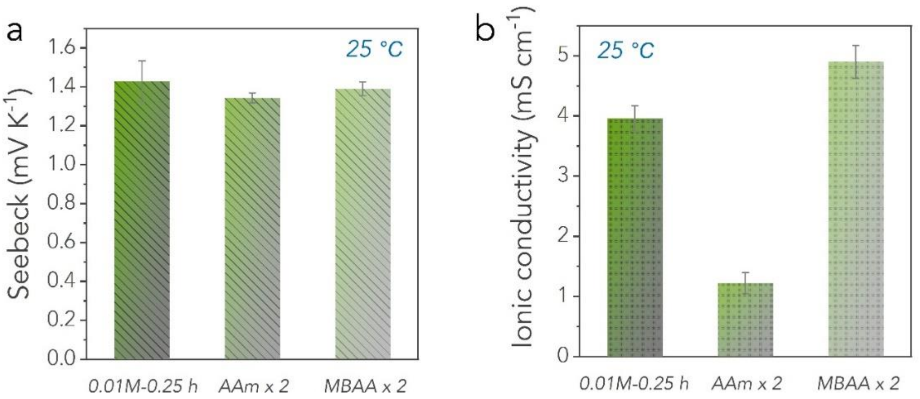

Fig. S9 a The Seebeck coefficient of the hydrogel samples with varied monomer content and crosslinker content. **b** The ionic conductivity of the hydrogel samples with varied monomer content and crosslinker content. "× 2" means the weight amount of monomer or crosslinker was doubled compared to the pristine sample. The Seebeck coefficient slightly decreased when either increasing the AAm content or increasing the degree of covalent crosslinking, which is possible due to the change of solvation effect of redox ions within the hydrogel. Meanwhile, the ionic conductivity substantially decreased when increasing the AAm content, as the water content was accordingly decreased. While increasing the degree of covalent crosslinking didn't notably affect the ionic conductivity, which differs from the case of increasing the degree of ionic crosslinking. This is because the pore size of the hydrogel is in the scale of micrometers, which can provide sufficient space for ion transport

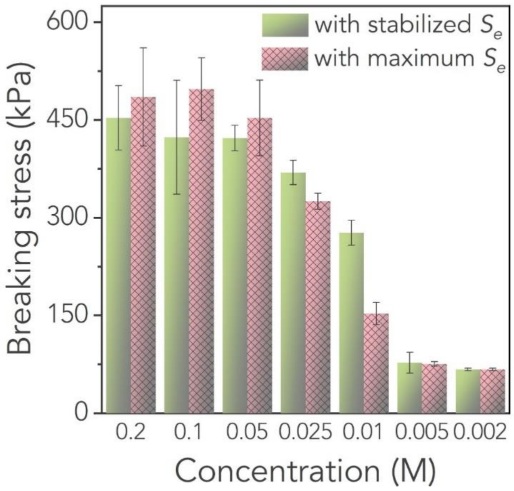

Fig. S10 Breaking stress of various PAAm/Fe-Alg hydrogels

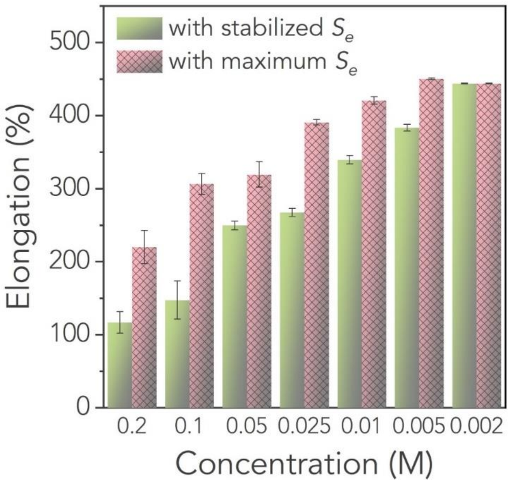

Fig. S11 Elongation at break of various PAAm/Fe-Alg hydrogels

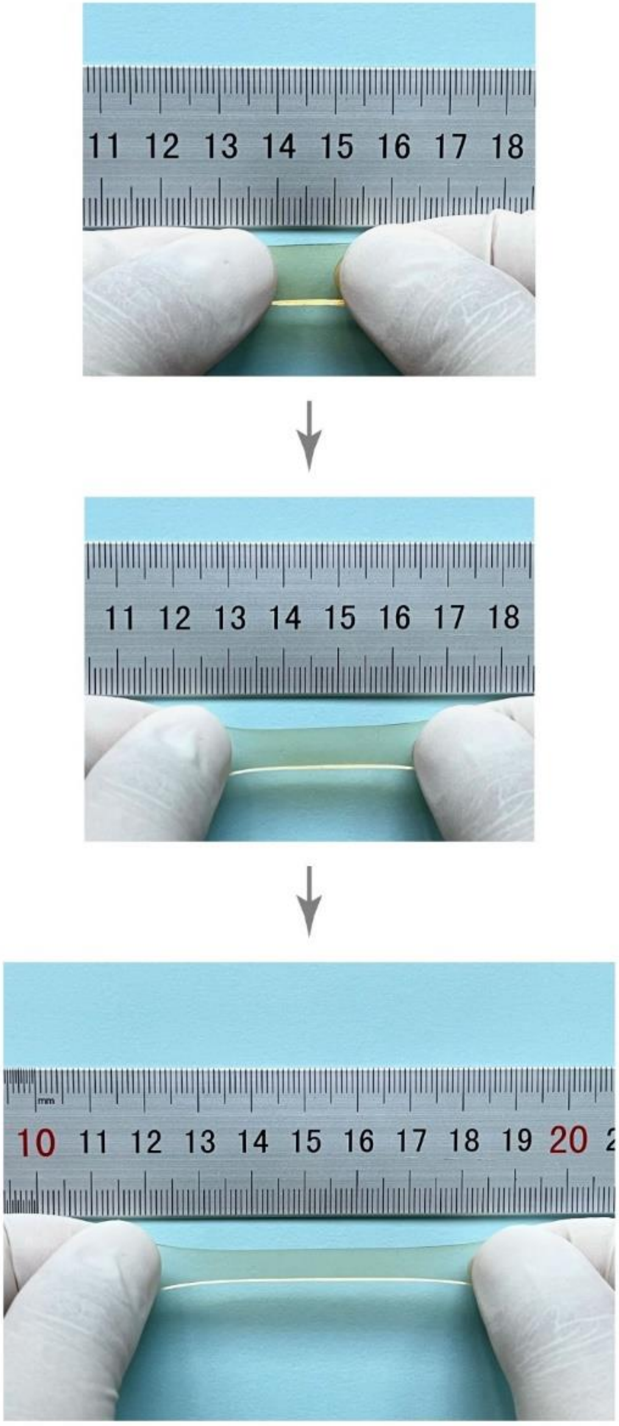

Fig. S12 Photos showing the stretching process of the 0.01 M-0.25 h PAAm/Fe-Alg hydrogel

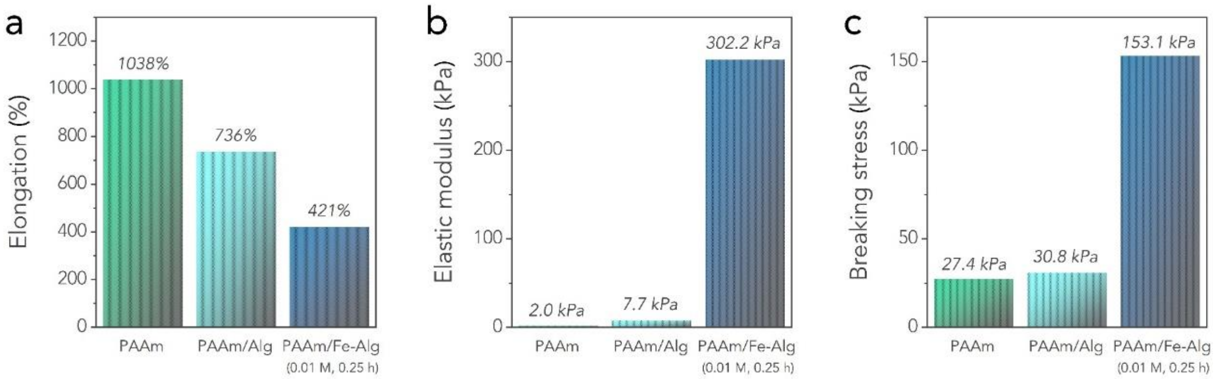

Fig. S13 Comparison of the mechanical properties of the pristine PAAm, PAAm/Alg and 0.01 M-0.25 h PAAm/Fe-Alg hydrogels

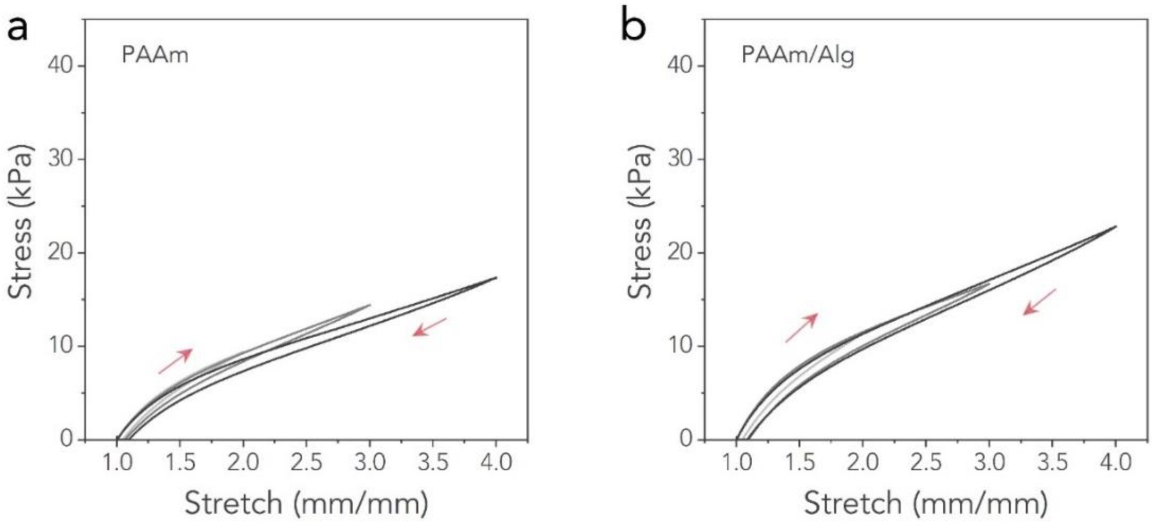

Fig. S14 Hysteresis loops of the pristine PAAm and PAAm/Alg hydrogels, which show neglectable hysteresis, *i.e.*, dissipated energy

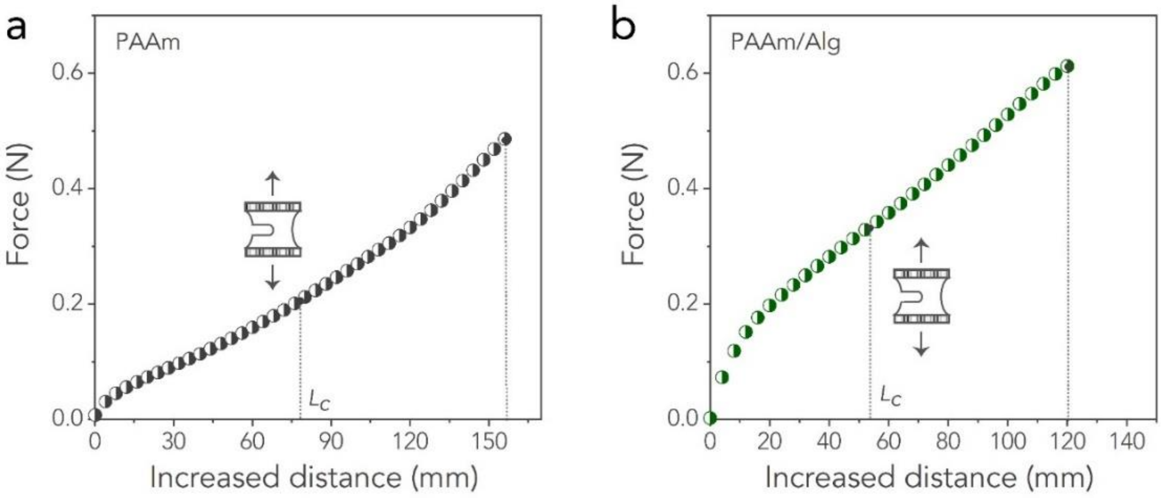

Fig. S15 Force-distance curves of the **a** pristine PAAm and **b** PAAm/Alg hydrogels for the calculation of facture toughness

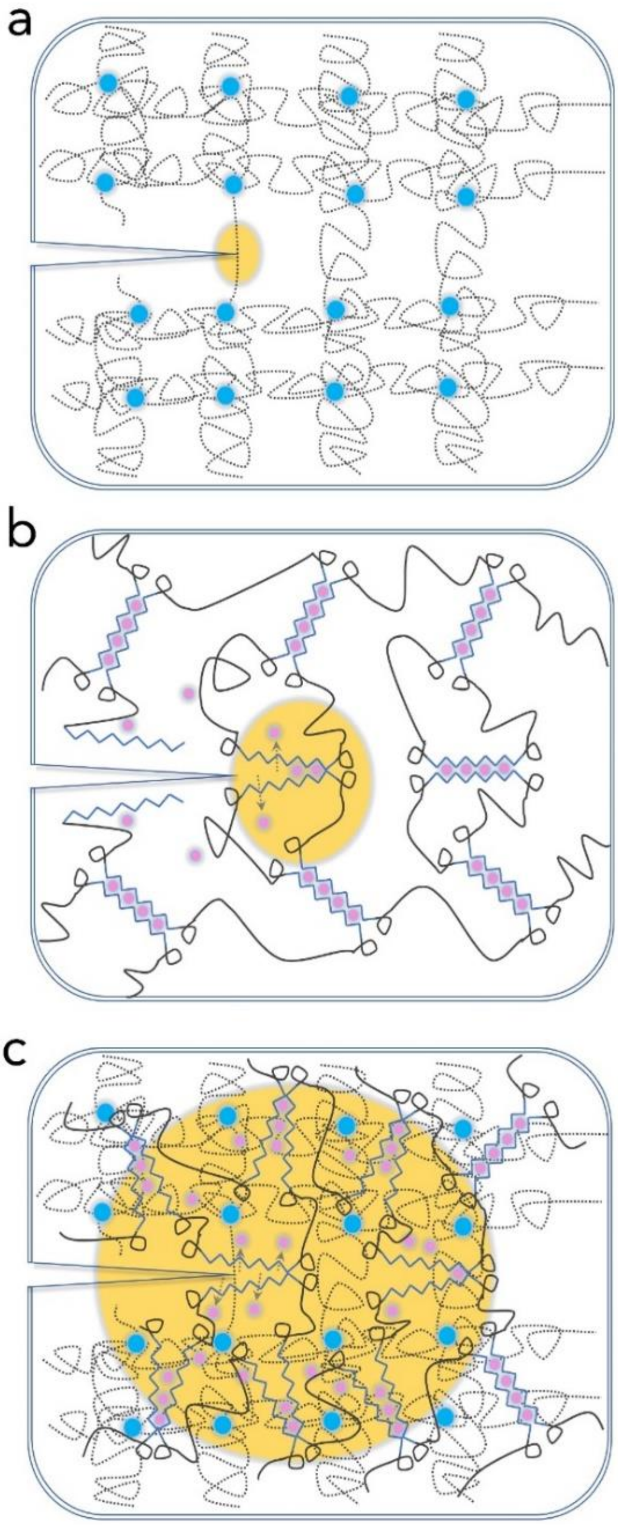

Fig. S16 Illustrative mechanism for the energy dissipation process. Blue dots represent covalent crosslinks, and purple dots represent ionic crosslinks. The yellow area represents the energy dissipation area. **a** Crack propagation in covalently crosslinked PAAm. **b** Crack propagation in ionically-crosslinked Alginate. **c** Crack propagation in dual-crosslinked PAAm/Fe-Alg

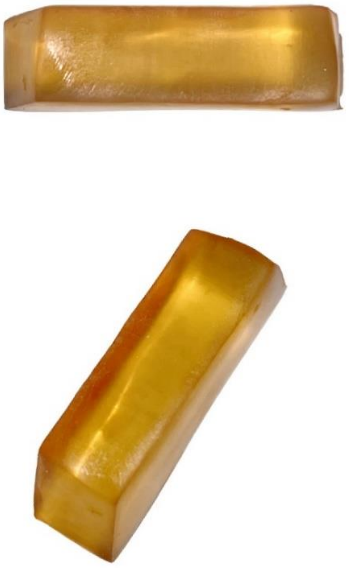

Fig. S17 Photos showing the smooth surface of the 0.01 M-0.25 h PAAm/Fe-Alg hydrogel after cut

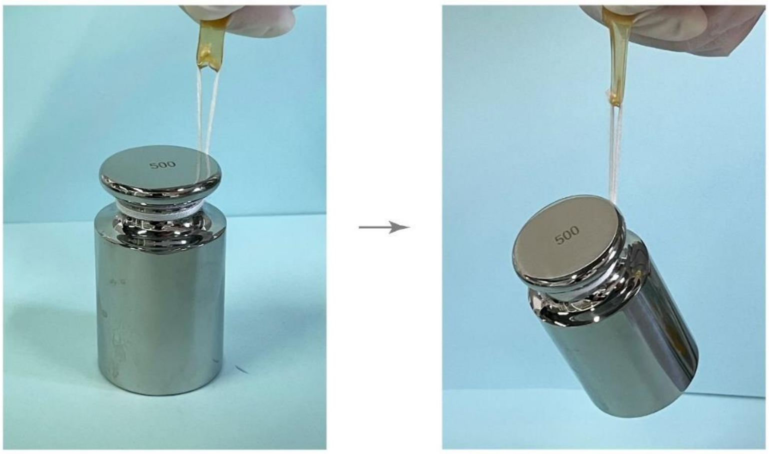

Fig. S18 Photos showing the thin 0.01 M-0.25 h PAAm/Fe-Alg hydrogel lifting a 500-g weight

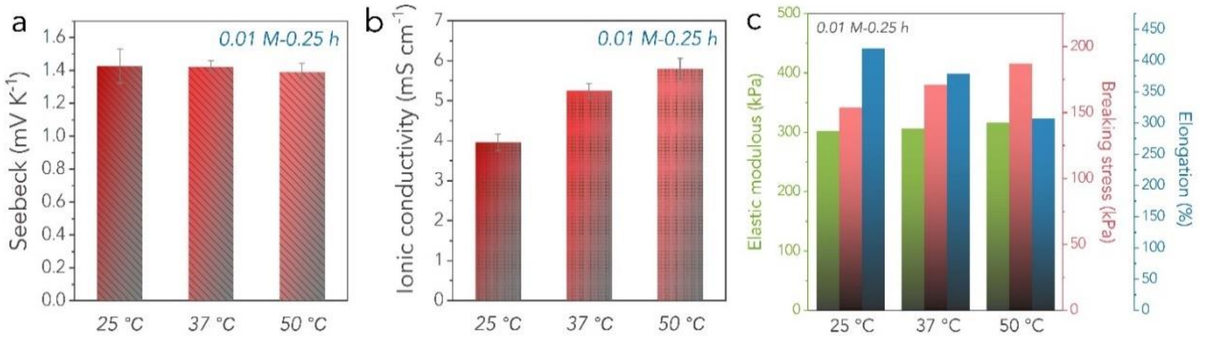

Fig. S19 **a** The Seebeck coefficient of the 0.01 M-0.25 h TEC measured under 25 °C (room temperature), 37 °C (body temperature) and 50 °C (high environmental temperature). It kept relatively stable under mild conditions, no notable variation was observed. **b** The ionic conductivity of the 0.01 M-0.25 h TEC measured under 25 °C, 37 °C and 50 °C. Obviously, the ionic conductivity gently increased with the increase of temperature. **c** The mechanical properties of the 0.01 M-0.25 h TEC measured under 25 °C, 37 °C and 50 °C. The mechanical properties were well sustained

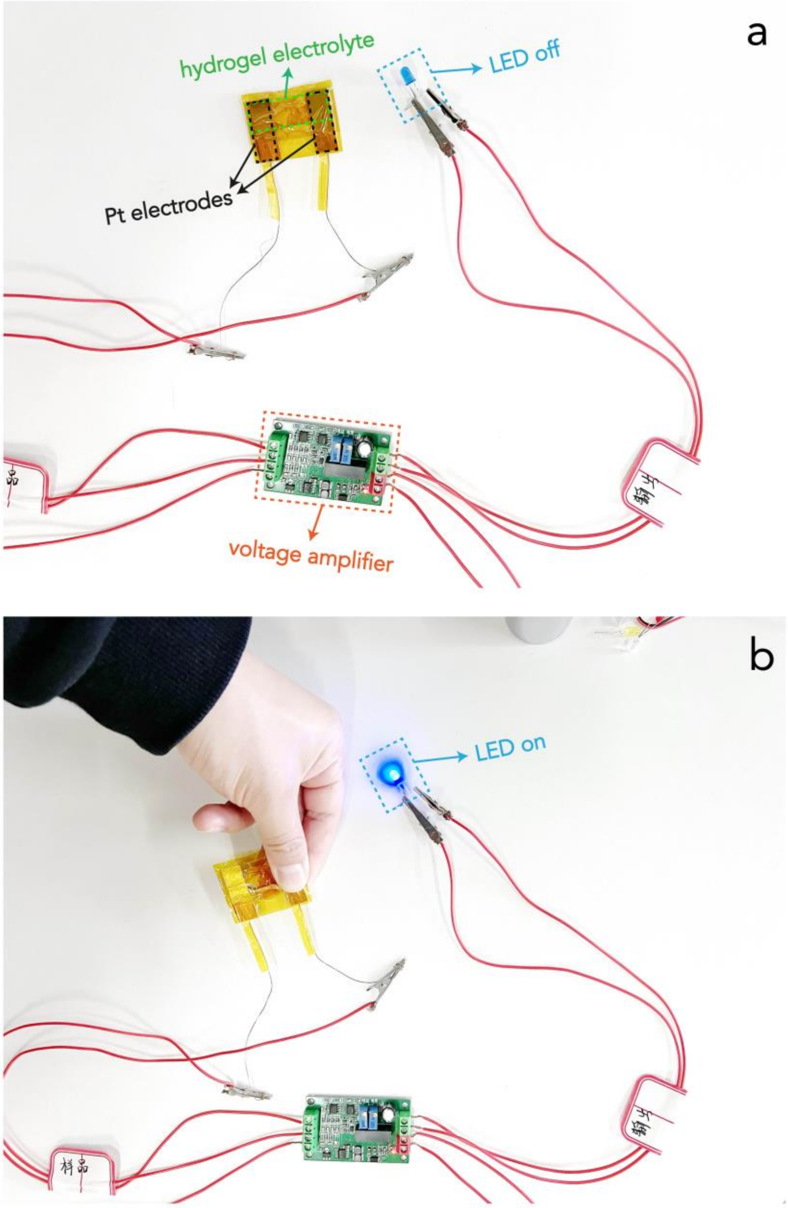

Fig. S20 The setup of the TEC to light up a LED. **a** The LED was off without a temperature gradient applied on the TEC. **b** The LED was on with fingers touching one side of the TEC that induced a temperature gradient

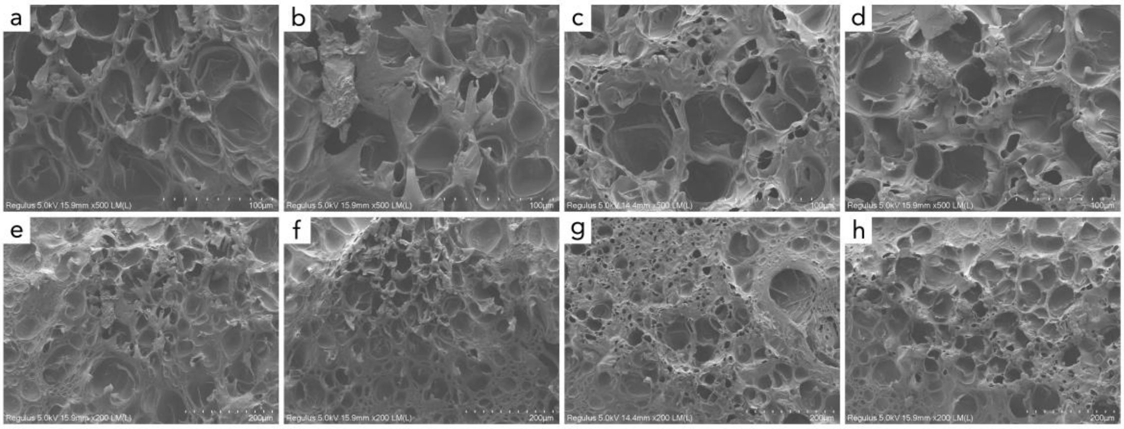

Fig. S21 SEM images of the hydrogel electrolytes after various mechanical operations including **a, e** stretching, **b, f** bending, **c, g** twisting and **d, h** hammering
